# Emergency Cardiac Surgery in Patients on Direct Oral Anticoagulants

**DOI:** 10.3389/fcvm.2022.884076

**Published:** 2022-04-13

**Authors:** Stefano De Paulis, Piergiorgio Bruno, Massimo Massetti

**Affiliations:** ^1^Department of Cardiovascular Sciences, Fondazione Policlinico Gemelli IRCCS, Rome, Italy; ^2^Università Cattolica del Sacro Cuore, Rome, Italy

**Keywords:** direct oral anticoagulants, cardiac surgery, cardiopulmonary bypass, bleeding, hemostasis

## Introduction

Cardiac surgery carries a high risk of perioperative bleeding and emergency surgery is one of the most significant factors in the determination of this risk. When perioperative bleeding ensues, blood products administration, and surgical re-exploration are generally required, with concomitant increase in perioperative morbidity and mortality (1). Bleeding risk is amplified in patients on anticoagulants and the expanding indication for direct oral anticoagulants (DOACs) has confronted perioperative physicians with new challenges related to the peculiar pharmacology of these agents. Guidelines for the management of bleeding patients taking DOACs are available but many uncertainties remain for their application to the cardiac surgery setting.

## Perioperative Bleeding in Cardiac Surgery

Emergency cardiac surgery has an inherently higher bleeding risk related to the pathology (aortic dissection, endocarditis), the intraoperative strategy (hypothermia) or the context (coronary revascularization after antiplatelet loading dose). Hemostatic balance during cardiac surgery is a rollercoaster ride: initial surgical dissection is best accomplished with a normal coagulation profile, cannulation and cardiopulmonary bypass require complete anticoagulation while final surgical hemostasis and the patient's course in the early post-operative period clearly benefit from complete restoration of hemostatic activity. Guidelines and consensus statements on the laboratory assessment of coagulation profile and optimal pharmacological treatment options have been released (2, 3). In this scenario the recent intake of therapeutic doses of DOACs poses additional burden on a complex task.

## Assessing Preoperative Coagulation Profile

Knowledge of the degree of anticoagulation determined by DOACs serum levels is important to decide the most appropriate treatment strategy. Standard coagulation tests like international normalized ratio (INR), prothrombin time (PT) and activated partial thromboplastin time (aPTT) have limited value because of the poor correlation with clinical hemostasis. Thrombin time is extremely sensitive to dabigatran, with significant influence even from subtherapeutic levels of drug. Standard thromboelastography/thromboelastometry assays are not sensitive enough to guide management, although newly developed tests that can detect DOACs are available but need clinical validation (4). Quantitative monitoring requires liquid chromatography/tandem mass spectrometry or, for direct thrombin inhibitors like dabigatran, an ecarin chromogenic assay or, for factor Xa inhibitors like apixaban, an anti-factor Xa activity assay (3). These quantitative tests are not readily available and have long turnaround time that makes them unavailable in an emergency setting.

## Treatment Options

Activated oral charcoal (50 g orally) can be given if DOAC was last ingested within 2–4 h to reduce residual drug absorption.

Different antidotes are available for the thrombin inhibitor and anti-factor Xa inhibitor DOACs. Idarucizumab is an anti-dabigatran monoclonal antibody fragment given as a 5 g initial dose (a second dose may be given if required) that rapidly corrects quantitative assays results even though rebound rise in clotting time after 12–24 h has been reported. Limited use in cardiac surgery before cardiopulmonary bypass has been described with no apparent thromboembolic complications (5). Andexanet alfa is a genetically modified factor Xa variant that prevents binding to factor Xa by all inhibitors (including low-molecular-weight heparin and fondaparinux). It is administered as a bolus dose followed by continuous infusion for up to 2 h and rapidly reduces anti-factor Xa activity. Andexanet alfa binds to heparin-antithrombin complexes preventing proper anticoagulation with heparin, so that alternative anticoagulation with bivalirudin has been suggested if the antidote is given before cardiopulmonary bypass (3).

Non-specific prohemostatic agents, such as four-factor prothrombin concentrate (4F-PCC), are a second line reversal strategy that has been associated with adequate bleeding control in DOACs-related bleeding (6). The dose ranges from 25 to 50 IU/kg and it can be used before cardiopulmonary bypass with no later interference with heparin anticoagulation.

During cardiopulmonary bypass hemoadsorption of apixaban with Cytosorb^®^ has been reported (it also binds antiplatelet drugs), while modified ultrafiltration could be effective only for dabigatran that has a low protein-bound fraction (7).

Supportive care with fresh frozen plasma, platelets, fibrinogen concentrate, desmopressin and antifibrinolytics, if necessary, should be provided in adherence with protocols for cardiac surgery (2) (Table 1).

**Table 1:** Treatment options.

		**Treatment**		
**Medication**	**Action**	**Before CPB**	**During CPB**	**After CPB**
Dabigatran	Direct thrombin inhibitor	•Activated charcoal •4F-PCC •Consider Idarucizumab	Hemofiltration	Supportive care for cardiac surgery
Apixaban, edoxaban, rivaroxaban, betrixaban	Factor Xa inhibitor	•Activated charcoal • 4F-PCC	Hemoadsorption	•Consider Andexanet-alfa •Supportive care for cardiac surgery

## Discussion

Guidelines have been established for patients on DOACs undergoing emergency non-cardiac surgery which recommend correction of hemostatic imbalance before skin incision (8). Cardiac surgery has the unique challenge of cardiopulmonary bypass that requires complete anticoagulation and at the same time aggravates the hemostatic profile via activation of coagulation and inflammatory cascade. Data on the safety and efficacy of DOACs antidotes given before cardiopulmonary bypass are scarce and heparin resistance has been reported with the use of andexanet alfa (9). Their use may be postponed to the end of surgery in case of persistent bleeding that is refractory to conventional coagulation supportive measures. Reports from the cardiac surgery literature indicate that pre-operative correction of hemostatic imbalance with 4F-PCC seems a reasonable initial approach that combines efficacy with safety and does not interfere with heparin anticoagulation during bypass (10). With the start of extracorporeal circulation, blood purification techniques may be instituted to enhance drugs removal. Hemofiltration can achieve significant clearance for dabigatran only while hemoadsorption with Cytosorb^®^ may be effective for removal of DOACs and antiplatelet agents.

One more thing to consider while managing anticoagulation during cardiopulmonary bypass is the fact that DOACs may interfere with activated clotting time (ACT) giving falsely low values that do not reflect actual anticoagulation (11).

The strategy of post-operative management should result from an assessment of the hemorragic and thromboembolic risks; as an example, the amount of chest tubes drainage that can be tolerated in the first post-operative hours. This would be a case-by-case assessment to decide how aggressive the correction of coagulopathy must be but also to choose timing and modality of post-operative anticoagulation that needs to be restarted.

## Author Contributions

All authors listed have made a substantial, direct, and intellectual contribution to the work and approved it for publication.

## Conflict of Interest

The authors declare that the research was conducted in the absence of any commercial or financial relationships that could be construed as a potential conflict of interest.

## Publisher's Note

All claims expressed in this article are solely those of the authors and do not necessarily represent those of their affiliated organizations, or those of the publisher, the editors and the reviewers. Any product that may be evaluated in this article, or claim that may be made by its manufacturer, is not guaranteed or endorsed by the publisher.
